# Application of K-Means Clustering Algorithm to Commercial Parameters of *Pleurotus* spp. Cultivated on Representative Agricultural Wastes from Province of Guayas

**DOI:** 10.3390/jof7070537

**Published:** 2021-07-04

**Authors:** Fabricio Guevara-Viejó, Juan Diego Valenzuela-Cobos, Purificación Vicente-Galindo, Purificación Galindo-Villardón

**Affiliations:** 1 Facultad de Ciencias e Ingeniería, Universidad Estatal de Milagro (UNEMI), 091050 Milagro, Ecuador; jguevarav@unemi.edu.ec (F.G.-V.); juan_diegova@hotmail.com (J.D.V.-C.); 2 Department of Statistics, University of Salamanca, 37008 Salamanca, Spain; purivic@yahoo.com

**Keywords:** K-means clustering algorithm, *Pleurotus ostreatus*, *Pleurotus djamor*, commercial parameters, visualization

## Abstract

Data of the commercial parameters of *Pleurotus ostreatus* and *Pleurotus djamor* were analyzed using the data mining technique: K-means clustering algorithm. The parameters evaluated were: biological efficiency, crop yield ratio, productivity rate, nutritional composition, antioxidant and antimicrobial activities in the production of fruit bodies of 50 strains of *Pleurotus ostreatus* and 50 strains of *Pleurotus djamor*, cultivated on the most representative agricultural wastes from the province of Guayas: 80% sugarcane bagasse and 20% wheat straw (M1), and 60% wheat straw and 40% sugarcane bagasse (M2). The database of the parameters obtained in experimental procedures was grouped into three clusters, providing a visualization of the strains with a higher relation to each parameter (vector) measured.

## 1. Introduction

The province of Guayas, comprising 25 cantons, including the canton of Guayaquil, which consists of the Municipality of Guayaquil and two areas of urban expansion, along with five rural cantons: Morro, Juan Gómez Rendón, Posorja, Puna and Tenguel. The province of Guayas is part of Zone 5, according to SENPLADES, National Secretariat for Planning and Development, which subdivides the country into nine zones. The main land-use of the Guayas province is agriculture (27%), followed by livestock production and aquaculture. This province has gone through three main economic booms [1].

The first economic boom occurred in 1880 when it was the main cocoa exporting city (between 20% and 25% of the world), becoming an important commercial and financial center, which generated an increase in the city’s population [2], the landowners monopolized the best land and access to transportation, as well as controlling key sources of credit and commercial links [3]. The second economic boom was in 1950 with the so-called “banana boom”, and foreign banana-producing companies arrived, such as the United Fruit Company in Tenguel, one of the largest banana plantations (localized 100 miles south of Guayaquil) [4]. The third boom was the oil boom in 1972 that brought new developments, mainly in the form of land invasions on the outskirts of the city, causing an immense deterioration of the agricultural sector. The lack of a national policy for small-scale rural agriculture led many of the rural small farmers (mainly indigenous from the central areas) to abandon their plots and engage in non-agricultural activities, most often in the urban informal sector [5]. With the previously described background, it is important to continue using the agricultural wastes that continue to be generated from all of the provinces of Guayas, having an innovative idea, such as the production of edible mushrooms, also called “vegetable steak” [6].

The cultivation of edible mushrooms has gradually grown using homemade techniques until becoming a highly technical industry [7,8,9,10]. The world production of edible mushrooms has grown in the last three years, with an annual increase of 24.5%. The nutritional value of edible mushrooms is high in comparison with other kinds of food. According to studies carried out by food specialists, mushrooms have a protein content between 19% and 35%, compared to vegetables (vegetables and fruits) that only have protein between 7.3% from 13.2%; on the other hand, milk, meat and eggs have a protein content between 25% and 90%. However, at the amino acid level, protein precursor substances, such as lysine and tryptophan, reach levels between 1.1 and 2.09 g. On the other hand, the low carbohydrate content makes mushrooms a low-energy food and is recommended as a dietary one. In addition, the content of essential fatty acids such as oleic and linoleic is found in appreciable quantities [11]. Edible mushrooms are nutritious plants that contain riboflavin, nicotinic acid, pantothenate and biotin, which lower blood pressure, prevent atherosclerosis and boost the immune system (immune system) against disease [12].

One of the principal genera of edible mushrooms with the highest production around the world is *Pleurotus* spp. [13,14]. These mushrooms are characterized by their nutritional value and are an important source of proteins, vitamins and minerals [15,16]. These species require tropical or subtropical climates similar to the Province of Guayas for the cultivation and production of fruiting bodies [17,18,19]. Additionally, these mushrooms are actively used in medical treatments with antioxidant and antimicrobial properties protecting health by damping active oxygen and free radicals [20]. However, the lack of knowledge of the nutritional and pharmaceutical properties of *Pleurotus* spp. has not been allowed to be used for the benefit of human health in Ecuador. In order to illustrate adequate visualization, the use of data mining tools, such as the K-means clustering algorithm, upon big data, about the commercial parameters of *Pleurotus* spp. is important [21].

In this paper, the focus is on clustering. The clusters have been applied in many research areas such as mathematics, engineering, economics, marketing, machine learning, pattern recognition, genetics, bioinformatics, psychology, biology, data compression and information retrieval [22]. The initial values of cluster “centroids” are randomly selected from the available data. Updating centroids and clustering of data is then repeated until convergence is reached or for a defined number of iterations. A new centroid for a cluster is calculated based on each data sample that belongs to that cluster, and the initial centroids are usually chosen randomly for the application of K-means-type algorithms [23].

The main goal was to use a data mining technique, the K-means clustering algorithm, to verify the influence of two mixtures of agricultural wastes obtained from the province of Guayas on the viability of the mushrooms production, the nutritional profile and also in the antioxidant and antimicrobial properties. The use of the K-means clustering algorithm allowed the indication of the strain cultivated on a specific mixture of agricultural wastes that obtained the highest values in commercial parameters. 

## 2. Materials and Methods

### 2.1. Mushroom Strains

In this study, 50 strains of *Pleurotus ostreatus* (PO) and 50 strains of *Pleurotus djamor* (PD) were used. These strains were collected from producers in the province of Guayas. The strains were maintained on MEA dishes and are deposited at the fungal collection of the Research and Development Laboratory of Ecuahidrolizados.

### 2.2. Substrate and Supplementation

Strains were cultivated using two mixtures of agricultural wastes: 80% sugarcane bagasse and 20% wheat straw (M1), and 60% wheat straw and 40% sugarcane bagasse (M2). The mixtures of agricultural wastes were moistened for 1 day. Subsequently, the mixture was placed (1 kg wet weight) in plastic bags and pasteurized for 10 h at 80 °C.

After pasteurization and conditioning, with the substrate at ambient temperature, the bags with the substrate were inoculated with 150 g of wheat grain previously colonized with the strains of *Pleurotus ostreatus* (PO) and the strains of *Pleurotus djamor* (PD). Thereafter, the bags with the substrate, were incubated in a dark room at a temperature of 30 ± 1 °C.

Finally, once the mycelium of the strain had colonized the substrate, the bags with the substrate were transferred to a room with favorable conditions for the fructification: relative humidity was maintained between 85% and 90%, a temperature of 25 ± 1 °C, air recirculation and period of illumination of 12 h [24].

### 2.3. Productivity Parameters

#### 2.3.1. Biological Efficiency

The biological efficiency (BE) is a productivity parameter that explains the capacity of the substrate to produce fruit bodies and was calculated using the following equation [25]:

(1) $$BE\left(\%\right)=\frac{fresh\;weight\;of\;mushrooms\;\left(g\right)}{weight\;of\;dry\;substrate\;\left(g\right)}\times 100$$

Equation (1). Biological efficiency of the mushrooms.

#### 2.3.2. Yield Ratio

The yield ratio is an analyzed variable widely adopted for industrial crops and was calculated with the following equation [26]:

(2) $$Y\left(\%\right)=\frac{fresh\;weight\;of\;mushrooms\;\left(g\right)}{fresh\;weight\;of\;substrate\;\left(g\right)}\times 100$$

Equation (2). Yield ratio of the mushrooms.

#### 2.3.3. Productivity Rate (PR)

The productivity rate is the relation between BE and the precocity (namely days between inoculation and harvest) and was calculated using the following equation [27]:

(3) $$PR\left(\%\;per\;day\right)=\frac{biological\;efficiency\;\left(\%\right)}{precocity\;\left(days\right)}$$

Equation (3). Productivity rate of the mushrooms.

### 2.4. Nutritional Composition

The nutritional value of the mushroom sample was analyzed using AOAC procedures concerning the composition of proteins, fat, carbohydrates and ash [28]. For the estimation of the crude protein content (N × 4.38), the macro-Kjeldahl method was used; the crude fat content was determined by extracting a known weight of sample with hexano, using a Soxhlet apparatus while the ash content was determined by calcination at 600 °C [29]. The total carbohydrate content (%C) was calculated by using the following equation:

(4) C(%)=100−(%moisture+%protein+%fat+%ash  contents)

Equation (4). Percentage of carbohydrates of the mushrooms.

### 2.5. Antioxidant Activity

To evaluate the antioxidant activity, the DPPH radical-scavenging assay was used. In the beginning, 30 μL of the extract and 270 μL of methanol containing DPPH radicals (6 × 10^−5^ mol L^−1^) were pipetted and mixed in a 96 well plate. The reaction mixture was incubated in the dark for 30 min, and the absorption was measured at 515 nm using a microplate reader [30]. The DPPH radical scavenging activity (RSA) was calculated as a percentage of DPPH discoloration using the following equation:

(5) $$RSA\left(\%\right)=\frac{ADPPH-AS}{ADPPH}\times 100$$

Equation (5). DPPH radical scavenging activity (RSA).

### 2.6. Antimicrobial Activity

The antimicrobial activity was analyzed using the following Gram-negative bacteria: *Pseudomonas aeruginosa* (ABN 187) and *Salmonella typhimurium* (ABN 572); and the following Gram-positive bacteria: *Micrococcus flavus* (ABP 147) and *Staphylococcus aureus* (ABP 784). The microorganisms are deposited at the Research and Development Laboratory of Ecuahidrolizados.

Bacterial suspensions were adjusted with sterile saline to a concentration of 1.0 × 10^6^ CFU/mL. The mushroom extracts of *Pleurotus* spp. were dissolved in 30% ethanol, mixed with nutrient media for bacteria (Tryptic Soy Broth) containing bacterial inoculum (1.0 × 10^5^ CFU per well) with a final volume of 100 µL [31].

### 2.7. Statistical Analysis

#### K-Means Clustering

The K-means grouping method is a non-hierarchical technique used to group observations into K groups. Each item is assigned to a group with the closest center. The algorithm iteratively updates the groups to minimize the variation of their elements. The basic K-means algorithm, which was used in this article, refers to the Euclidean metric to define the distance between the elements and the centers of the clusters [32]. The Euclidean distance is selected as the similarity index, and the clustering targets minimize the sum of the squares of the various types; that is, it minimizes [33]:

$$d=\overset{k}{\underset{k=1}{\sum }}\overset{k}{\underset{i=1}{\sum }}{∥(x_{i}-u_{k})∥}^{2}$$

where k represents K cluster centers, u_k_ represents the kth center, and x_i_ represents the ith point in the data set. The solution to the centroid u_k_ is as follows:

$$\frac{\partial }{\partial u_{k}}=\frac{\partial }{\partial u_{k}}\;\overset{k}{\underset{k=1}{\sum }}\overset{n}{\underset{i=1}{\sum }}{\left(x_{i}-u_{k}\right)}^{2}$$

$$=\overset{k}{\underset{k=1}{\sum }}\overset{n}{\underset{i=1}{\sum }}\frac{\partial }{\partial u_{k}}{\left(x_{i}-u_{k}\right)}^{2}$$

$$=\overset{n}{\underset{i=1}{\sum }}2\left(x_{i}-u_{k}\right)$$

$$u_{k}=\frac{1}{n}\overset{n}{\underset{i=1}{\sum }}x_{i}$$

Furthermore, the result of the K-means method is highly dependent on the number of clusters defined beforehand. In general, the iterative clustering method of K-means is implemented as follows: Step 1: A value of K is chosen. It is used as the initial set of K centroids. Step 2: Each of the objects is assigned to the group with the closest centroid. Step 3: The new centroids of the K groups are determined, calculating the mean of the group members. Step 4: Steps 3 and 4 are repeated until there are no changes in the criterion function after one iteration [34].

The main advantages of the K-means algorithm are its low complexity, it is computationally fast, the ability to handle large data sets and the flexibility to adjust the cluster number. K-means clustering was used to extract clusters from the dataset that had been optimized by feature selection.

Additionally, a PCA biplot [35] was applied to explore and visualize the different parameters and the most relevant responses.

## 3. Results and Discussion

The focus of this work was to determine the viability of the use of agricultural wastes from the province of Guayas on the cultivation of the strains *Pleurotus ostreatus* and *Pleurotus djamor* and assess its influence on commercial parameters: biological efficiency, crop yield ratio, productivity rate, nutritional composition, antioxidant and antimicrobial activities.

The numeration of the strains cultivated on the two mixtures of agriculture was made using the following distribution:

1–50: Strains of *Pleurotus ostreatus* or *Pleurotus djamor* cultivated on the mixture M1.

51–100: Strains of *Pleurotus ostreatus* or *Pleurotus djamor* cultivated on the mixture M2.

### 3.1. Productivity Parameters

**Figure 1:**
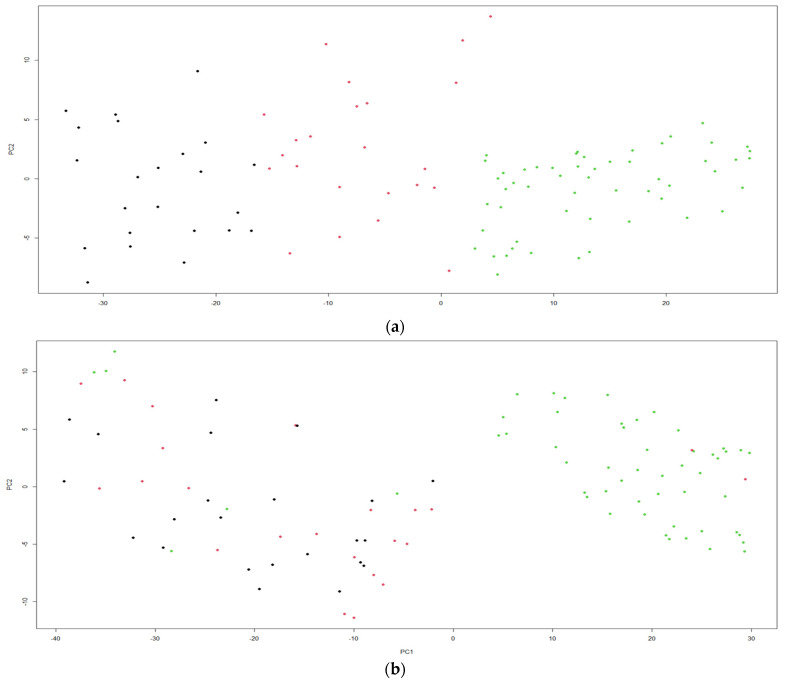
(**a**) K-means using 3 clusters for productivity parameters of *Pleurotus ostreatus* cultivated on two mixtures of agricultural wastes, (**b**) K-means using 3 clusters for productivity parameters of *Pleurotus djamor* cultivated on two mixtures of agricultural wastes.

Figure 1 shows the application of the K-means clustering algorithm method to 100 objects having three variables, with each one using the software RStudio. The graphic (a) presents the use of three clusters for the productivity parameters of *Pleurotus ostreatus* strains cultivated on agricultural wastes from the province of Guayas, while in the graphic (b) the use of three clusters for the productivity parameters of *Pleurotus djamor* strains grown on the two mixtures of substrates was shown. The results show the normal distribution of 100 data points around three clusters in each graphic. The size of each cluster is related to the number of data points, in graphic (a): the size of Cluster 1 (color red) is 34, the size of Cluster 2 (color black) is 27, and the size of Cluster 3 (color green) is 39. *Pleurotus ostreatus* strains grown on the two mixtures belonging to Cluster 3 did not show a relationship with the *Pleurotus ostreatus* strains grown on the two mixtures belonging to Cluster 1 and Cluster 2. This result indicates that the strains that belong to Cluster 1 and Cluster 2 showed higher values of the productivity parameters in comparison to the other *Pleurotus ostreatus* strains (Cluster 3). On the other hand, in graphic (b): the size of Cluster 1 (color red) is 34, the size of Cluster 2 (color red) is 27, and the size of Cluster 3 (color black) is 39. *Pleurotus djamor* strains produced on the two mixtures belonging to Cluster 3 did not show a relationship with the *Pleurotus djamor* strains cultivated on the two substrates belonging to Cluster 1 and Cluster 2. This result indicates that the strains belonging to Cluster 1 and Cluster 2 presented higher values of productivity parameters in comparison to *Pleurotus djamor* strains belonging to Cluster 3. Since the data points are normally distributed, the clusters vary in size with the maximum data points and minimum data points. The supplementation of the substrate on mushroom cultivation has been carried out with relative success, aiming at controlling pests or increasing crop yields [36]. The results of the productivity parameters obtained were influenced by the different strains and the mixtures used in the research.

**Figure 2:**
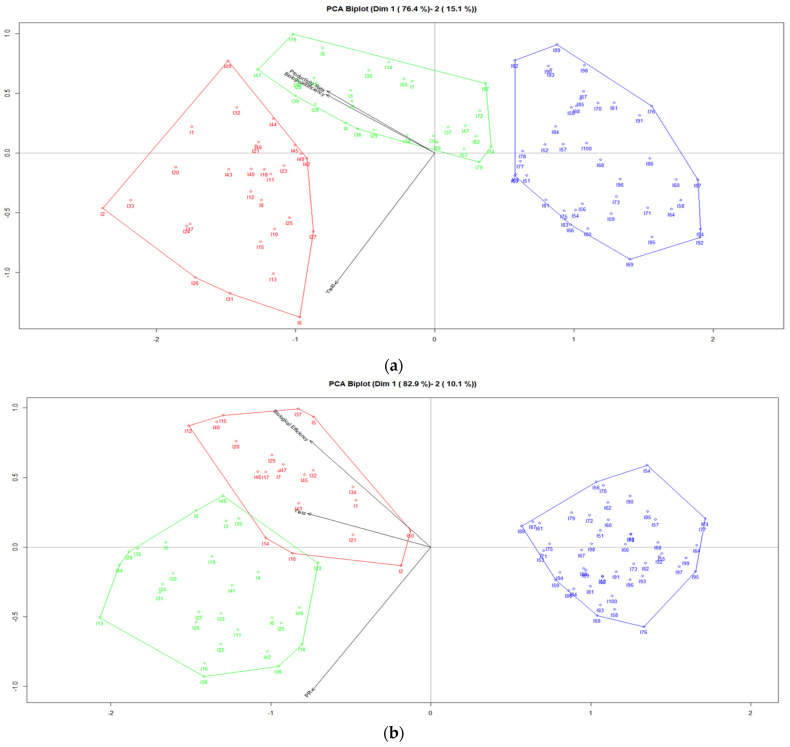
(**a**) PCA Biplot for productivity parameters of *Pleurotus ostreatus*, (**b**) PCA Biplot for productivity parameters of *Pleurotus djamor*.

Figure 2 shows the factorial graph of the plane 1–2 (PCA Biplot). Graphic (a) presents the accumulated inertia amounts to 91.5%, while graphic (b) presents the accumulated inertia amounts to 93.0. In addition, clusters have been calculated using the Biplot coordinates; the overview of clusters is based on three variables. In graphic (a), we observe important differences between clusters, Cluster 2 (color green) indicates the presence of 29 strains of *Pleurotus ostreatus* cultivated on the two mixtures of agricultural wastes with a higher relation to biological efficiencies and production rates, while Cluster 1 (color red) indicates the presence of 28 strains of *Pleurotus ostreatus* cultivated on the two mixtures of substrates with a higher relation to the yields, and Cluster 3 (color blue) indicates the presence of 43 strains of *Pleurotus ostreatus* cultivated on the two mixtures of agricultural wastes. On the other hand, in graphic (b) also there are differences between the clusters, Cluster 1 (color red) indicates the presence of 22 strains of *Pleurotus djamor* growth on the two mixtures of agricultural wastes with a higher relation to biological efficiencies and yields, whereas Cluster 2 (color green) indicates the presence of 28 strains of *Pleurotus djamor* cultivated on the two mixtures of substrates with a higher relation to production rates, and Cluster 3 (color blue) indicates the presence of 50 strains of *Pleurotus djamor* growth on the two mixtures of substrates.

The commercial production of mushrooms is largely determined by the availability and utilization of cheap materials of agricultural wastes that represent the ideal and most promising substrates for cultivation [37,38]. The use of these agricultural wastes from the province of Guayas can be used to obtain the highest productivity of fruit bodies providing an alternative for the mushroom market.

### 3.2. Nutritional Composition and Biological Properties

**Figure 3:**
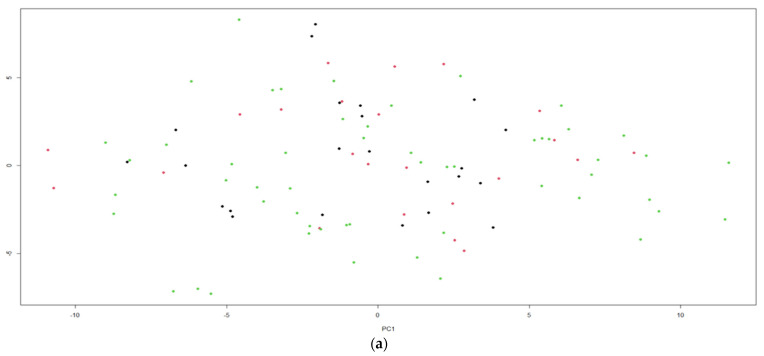

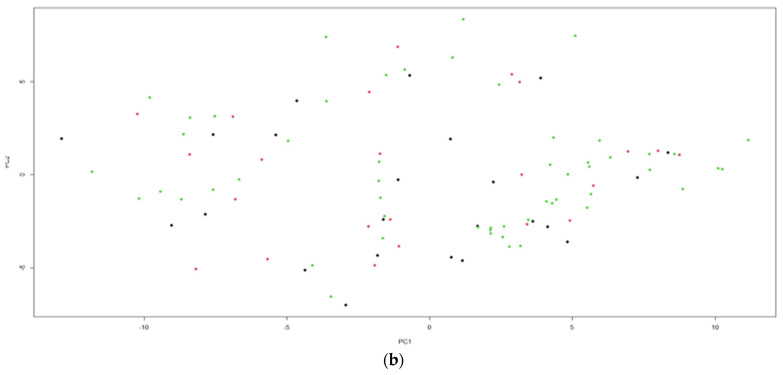
(**a**) K-means using 3 clusters for biological properties of *Pleurotus ostreatus* cultivated on two mixtures of agricultural wastes, (**b**) K-means using 3 clusters for biological properties of *Pleurotus djamor* cultivated on two mixtures of agricultural wastes.

Figure 3 presents the use of method K-means clustering algorithm to 100 objects having seven variables, each one using the software RStudio. Graphic (a) shows the application of three clusters for the nutritional composition and biological properties of *Pleurotus ostreatus* fruit bodies produced on agricultural wastes from the province of Guayas, while in graphic (b) the use of three clusters for the nutritional composition and biological properties of *Pleurotus djamor* mushrooms cultivated on the two mixtures of substrates is shown. A normal distribution of 100 data points around three clusters in each graphic was presented. The size of each cluster is related to the number of data points, in graphic (a): the size of Cluster 1 (color red) is 23, the size of Cluster 2 (color black) is 23, and the size of Cluster 3 (color green) is 54. The three clusters present the *Pleurotus ostreatus* fruit bodies with the highest values of nutritional composition and biological properties. On the other hand, in graphic (b): the size of Cluster 1 (color red) is 23, the size of Cluster 2 (color black) is 23, and the size of Cluster 3 (color green) is 54. *Pleurotus djamor* mushrooms with the highest values of nutritional composition and biological properties are shown by the three clusters. It is important to indicate that the clusters vary in size with maximum data points and minimum data points.

**Figure 4:**
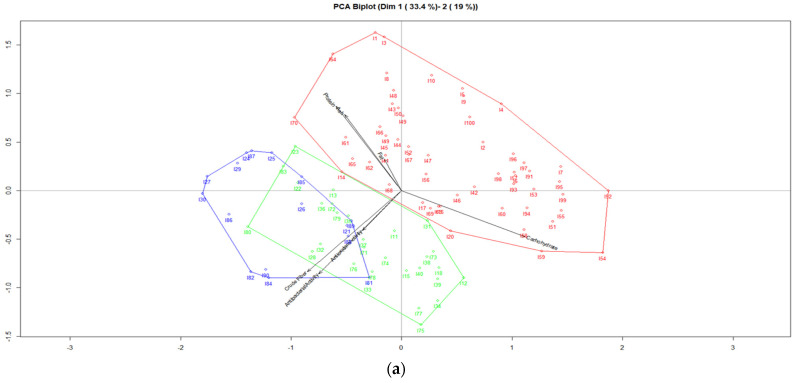

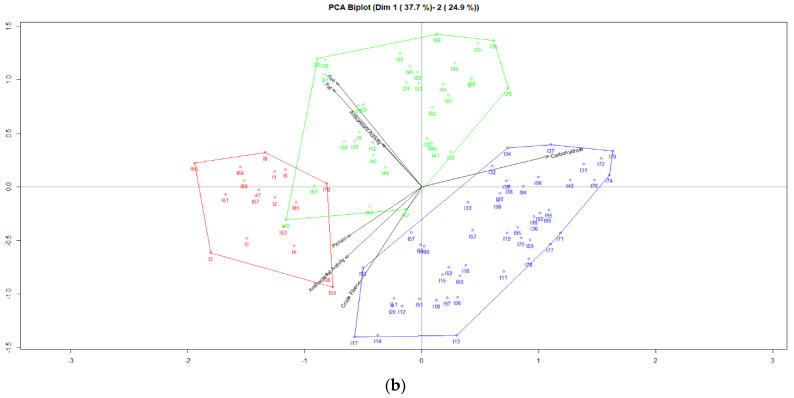
(**a**) PCA Biplot for biological properties of *Pleurotus ostreatus*, (**b**) PCA Biplot for biological properties of *Pleurotus djamor*.

Figure 4 shows the PCA Biplot of the plane 1–2, graphic (a) indicates the accumulated inertia amounts to 52.4%, while graphic (b) presents the accumulated inertia amounts to 62.6%. The three clusters have been calculated using the Biplot coordinates, the overview of clusters is based on seven variables. Graphic (a) shows important differences between clusters, Cluster 1 (color blue) indicates the presence of fruit bodies of 16 strains of *Pleurotus ostreatus* cultivated on the two mixtures of agricultural wastes with a higher relation to the crude fiber contents and antibacterial activities, while Cluster 2 (color green) indicates the presence of mushrooms of 28 strains of *Pleurotus ostreatus* cultivated on the two mixtures of food wastes with a higher relation to the antioxidant activities, and Cluster 3 (color red) indicates the presence of fruit bodies of 56 strains of *Pleurotus ostreatus* cultivated on the two mixtures of agricultural wastes with a higher relation to the protein, ash, fat and carbohydrate contents. On the other hand, in graphic (b), there are also differences between the clusters, Cluster 1 (color red) indicates the presence of 19 strains of mushrooms of *Pleurotus djamor* growth on the two mixtures of agricultural wastes with a higher relation to protein contents and antibacterial activities, whereas Cluster 2 (color green) indicates the presence of fruit bodies of 34 strains of *Pleurotus djamor* cultivated on the two mixtures of substrates with a higher relation to ash and fat contents and also antioxidant activities, and Cluster 3 (color blue) indicates the presence of mushrooms of 47 strains of *Pleurotus djamor* growth on the two mixtures of substrates with a higher relation to carbohydrate and crude fiber contents.

The moisture and fat contents of the mushrooms are influenced by the composition of the agricultural wastes used in the cultivation of edible fungi [39,40]. The nutritional composition of the mushrooms is influenced by the strains of the edible fungi and also by the agricultural wastes used in the cultivation, so we indicate based on the results that the food wastes from the province of Guayas can be used to produce fruit bodies with the highest biological properties.

## 4. Conclusions

The K-means clustering algorithm was used to obtain proper grouping data using three clusters and providing visualization about the relationships between strains of edible fungi *Pleurotus ostreatus* and *Pleurotus djamor* cultivated on the most representative agricultural wastes from the province of Guayas, with the commercial parameters measured in experimental procedures.

PCA Biplots presented that the use of mixture 1 in the cultivation of the strains of edible fungi *Pleurotus ostreatus* and *Pleurotus djamor* has a higher relation to the productivity parameters: biological efficiencies, crop yields and productivity rates.

The use of the K-means clustering algorithm on the commercial parameters of edible fungi *Pleurotus ostreatus* and *Pleurotus djamor*, cultivated on two mixtures of agricultural wastes, allowed the indication of how to obtain the highest values in productivity parameters or biological properties due to the strain grown on a specific substrate.

## Data Availability

Not applicable.
